# Ketone supplementation abolished isoflurane anesthesia-induced elevation in blood glucose level and increased recovery time from anesthesia in Wistar Albino Glaxo Rijswijk rats

**DOI:** 10.1186/s12871-023-02000-8

**Published:** 2023-02-07

**Authors:** Zsolt Kovács, Dominic P. D’Agostino, Csilla Ari

**Affiliations:** 1 Savaria Department of Biology, ELTE Eötvös Loránd University, Savaria University Centre, Szombathely, Hungary; 2 Ketone Technologies LLC, Tampa, FL USA; 3 Department of Molecular Pharmacology and Physiology, Laboratory of Metabolic Medicine, Morsani College of Medicine, University of South Florida, Tampa, FL USA; 4 Institute for Human and Machine Cognition, Ocala, FL USA; 5 Department of Psychology, Behavioral Neuroscience Research Laboratory, University of South Florida, 4202 E. Fowler Ave, PCD 3127, Tampa, FL USA

**Keywords:** Isoflurane anesthesia, Ketone supplement, Ketosis, Glucose, Recovery time, WAG/Rij rat

## Abstract

**Background:**

It has been suggested that administration of exogenous ketone supplements (EKSs) not only increases blood ketone body levels but also decreases blood glucose level and modulates isoflurane-induced anesthesia in different rodents, such as Wistar Albino Glaxo Rijswijk (WAG/Rij) rats. Thus, we investigated whether administration of EKSs can modulate the isoflurane anesthesia-generated increase in blood glucose level and the time required to recover from isoflurane-induced anesthesia.

**Methods:**

To investigate the effect of EKSs on isoflurane anesthesia-induced changes in blood glucose and R-β-hydroxybutyrate (R-βHB) level as well as recovery time from anesthesia, we used KEMCT (mix of ketone ester/KE and medium chain triglyceride/MCT oil in a 1:1 ratio) in WAG/Rij rats. First, to accustom the animals to the method, water gavage was carried out for 5 days (adaptation period). After adaptation period, rats of first group (group 1) were gavaged by water (3 g/kg), whereas, in the case of second group (group 2), the diet of animals was supplemented by KEMCT (3 g/kg, gavage) once per day for 7 days. One hour after the last gavage, isoflurane (3%) anesthesia was induced for 20 min (group 1 and group 2) and the time required for recovery from anesthesia was measured by using righting reflex. Subsequently, blood levels of both R-βHB and glucose were also evaluated. Changes in blood glucose and R-βHB levels were compared to control, which control glucose and R-βHB levels were measured on the last day of the adaptation period (group 1 and group 2). Time required for recovery from isoflurane anesthesia, which was detected after 7^th^ KEMCT gavage (group 2), was compared to recovery time measured after 7^th^ water gavage (group 1).

**Results:**

The KEMCT maintained the normal glucose level under isoflurane anesthesia-evoked circumstances preventing the glucose level elevating effect of isoflurane. Thus, we demonstrated that administration of KEMCT not only increased blood level of R-βHB but also abolished the isoflurane anesthesia-generated increase in blood glucose level. Moreover, the time required for recovery from isoflurane-evoked anesthesia increased significantly in KEMCT treated animals.

**Conclusions:**

Putative influence of elevated blood ketone body level on isoflurane-evoked effects, such as modulation of blood glucose level and recovery time from anesthesia, should be considered by anesthesiologists.

## Background

Inhalational (volatile) anesthetics, such as isoflurane (1-chloro-2,2,2-trifluoroethyl difluoromethyl ether) can easily cross the blood–brain barrier and neuronal membrane and are able to evoke general anesthesia [1–3]. Under isoflurane-evoked anesthesia, blood glucose level was increased likely through both impaired glucose clearance and increased glucose production [4, 5]. It has also been demonstrated that hyperglycemic effect of isoflurane anesthesia may generate different side effects [6–9].

Exogenous ketone supplements (EKSs), such as ketone salts (KSs) and ketone esters (KEs), as well as their mix with medium chain triglycerides (MCTs; e.g. KEMCT) are able to increase and maintain blood level of ketone bodies (e.g., β-hydroxybutyrate/βHB) and decrease blood glucose level in animal models and human [10–13]. It has been suggested that EKSs can modulate not only sleep, but also sleep-like effects and isoflurane-induced anesthesia [14–16]. Indeed, EKSs, such as KE, KS and KEMCT delayed the onset of isoflurane-induced light phase of anesthesia (immobility) in rodents, such as WAG/Rij (Wistar Albino Glaxo Rijswijk) rats [17]. This last influence was modulated likely by EKSs-generated increase in adenosine levels, thereby increase in activity of adenosine A1 receptors (A1Rs) [18]. Moreover, it was also suggested that A1Rs could increase the time required for recovery from inhalational anesthesia [19, 20] and insulin sensitivity [21, 22]. Consequently, theoretically, administration of EKSs may modulate both the time required for recovery from isoflurane anesthesia and isoflurane-induced increase in blood glucose level. However, influence of EKSs on the time required for recovery from isoflurane anesthesia (emergence from anesthesia) and isoflurane anesthesia-generated increase in blood glucose level were not investigated yet. 

Righting reflex is a postural response of animals evoked by sensory signals from visual, vestibular and somatosensory systems and carried out through reticulospinal and vestibulospinal tracts [23]. By this reflex, animals can reorient themselves when placed on their back or side (i.e. their paws/feet will be oriented towards the ground again) [1]. Loss of righting reflex is used as a behavioral indicator of general anesthesia, whereas recovery (return) of righting reflex is an indicator of emergence from anesthesia in animal studies [1, 23, 24]. Thus, building on our previous studies on EKSs-evoked effects on isoflurane anesthesia-induced influences in a well-established model of human absence epilepsy WAG/Rij rat [17, 18, 25], we investigated the effect of KEMCT administration on both the time required for recovery from isoflurane anesthesia by using righting reflex and isoflurane anesthesia-generated changes in blood glucose levels in WAG/Rij rats.

## Methods

### Animals

Treatments of animals were carried out based on the Hungarian Act of Animal Care and Experimentation (1998, XXVIII, Sect. 243), European Communities Council Directive (86/609/EEC) and EU Directive 2010/63/EU. The experiments were approved by the Animal Care and Experimentation Committee of the Eötvös Loránd University (Savaria University Centre) and National Scientific Ethical Committee on Animal Experimentation (Hungary) under license number VA/ÉBÁF-ÁO/00279–4/2021.

Male WAG/Rij rats (*n* = 24; 10 months old, 302–331 g; breeding colony of WAG/Rij rats: Eötvös Loránd University, Savaria University Centre) were housed in groups (4 animals in a group) under standard laboratory conditions (12:12 h light—dark cycle; light was on between 08.00 AM and 08.00 PM). We provided free access to food and water and air-conditioned room at 22 ± 2 °C. The rats were euthanized after the day of the last treatment by isoflurane. All efforts were made to minimize not only pain and suffering, but also the number of animals used.

### Treatment groups and design of experiments

KE (1,3-butanediol – acetoacetate diester) was developed by D’Agostino et al. [11] (University of South Florida, USA) and Savind, Inc. (Urbana, USA) whereas MCT oil (containing approximately 60% caprylic triglyceride and 40% capric triglyceride) was purchased from Now Foods (Bloomingdale, IL, United States).

We demonstrated previously the tolerability and effectiveness of KEMCT (mix of KE and MCT oil in a 1:1 ratio) given by intragastric gavage [18, 26–28]. KEMCT administered once per day by intragastric gavage for 7 days effectively induced ketosis in our previous studies [18, 27, 28]. Therefore, in this study, rats were fed with standard rodent chow diet (ad libitum access to normal rat chow) and received 3.0 g/kg body weight/day KEMCT by gavage for 7 days.

Similar to our previous studies [17, 29, 30], the 7 days gavage treatment was preceded by water gavage for 5 days to accustom the animals to gavage. After this adaptation period, animals were randomly assigned into 2 groups with 12 animals in each group. Animals of first group (group 1) were gavaged by water (3 g/kg), whereas rats of second group (group 2) were gavaged by KEMCT (3 g/kg) once per day for 7 days. To generate anesthesia, isoflurane-air mixture (3% isoflurane in isoflurane – air mixture) was used as previously described [17, 18, 24]. One hour after the last (7^th^) gavage, isoflurane anesthesia was induced in an airtight anesthesia chamber for 20 min (group 1 and group 2) to ensure enough time to both loss of righting reflex and develop significant increase in blood glucose level by isoflurane [5]. After 20 min, the time required for recovery (emergence) from isoflurane-evoked anesthesia was measured by using righting reflex [1, 24]. Righting reflex was tested by placing rats on their backs in the middle of a Plexiglas box (100 × 60 cm with 40 cm high wall) immediately after termination of isoflurane anesthesia. When all paws of animals were oriented towards the ground it was considered that rats were recovered from isoflurane-generated anesthesia (recovery time: the time from the termination of isoflurane anesthesia to recovery from righting loss). Experiments were carried out in the daytime at the room temperature (22 ± 2 °C) [24]. Recovery process was video-recorded by a blinded observer.

### Detection of blood R-βHB and glucose levels and measurement of body weight

Blood was taken from the tail vein of rats. A commercially available glucose and ketone monitoring system (Precision Xtra™, Abbott Laboratories, USA) was used for monitoring of blood glucose and R-βHB levels [10, 29]. Control glucose and R-βHB levels were measured on the last (5^th^) day of the adaptation period 90 min after the gavage (control; group 1 and group 2). Moreover, glucose and R-βHB levels were also measured 90 min after the last (7^th^) water gavage combined with isoflurane anesthesia (group 1; on awake animals, several min after recovery from anesthesia), first KEMCT gavage (group 2) and last (7^th^) KEMCT gavage combined with isoflurane anesthesia (group 2; on awake animals, several min after recovery from anesthesia).

Body weight of rats were measured on last (5^th^) day of the adaptation period (control) and after the last (7^th^) day of water (group 1) or KEMCT (group 2) gavage.

### Statistics

All data were presented as the mean ± standard error of the mean (S.E.M.). Blood glucose and R-βHB levels and body weight on last (7^th^) day of gavage were compared to control (control levels were measured on the last/5^th^ day of the adaptation period; group 1 and group 2). Time required for recovery (recovery time) from isoflurane anesthesia, which was detected after 7^th^ KEMCT gavage combined with isoflurane anesthesia (group 2), was compared to recovery time measured after 7^th^ water gavage and isoflurane anesthesia (group 1). GraphPad Prism version 9.2.0 (using a two-way ANOVA with Tukey’s multiple comparisons test and Šídák's multiple comparisons test and t-test) was used for data analysis [17]. Results were considered significant when *p* < 0.05.

## Results

### Effect of KEMCT treatment on isoflurane anesthesia-induced changes in blood glucose and R-βHB levels and on body weight

**Fig. 1:**
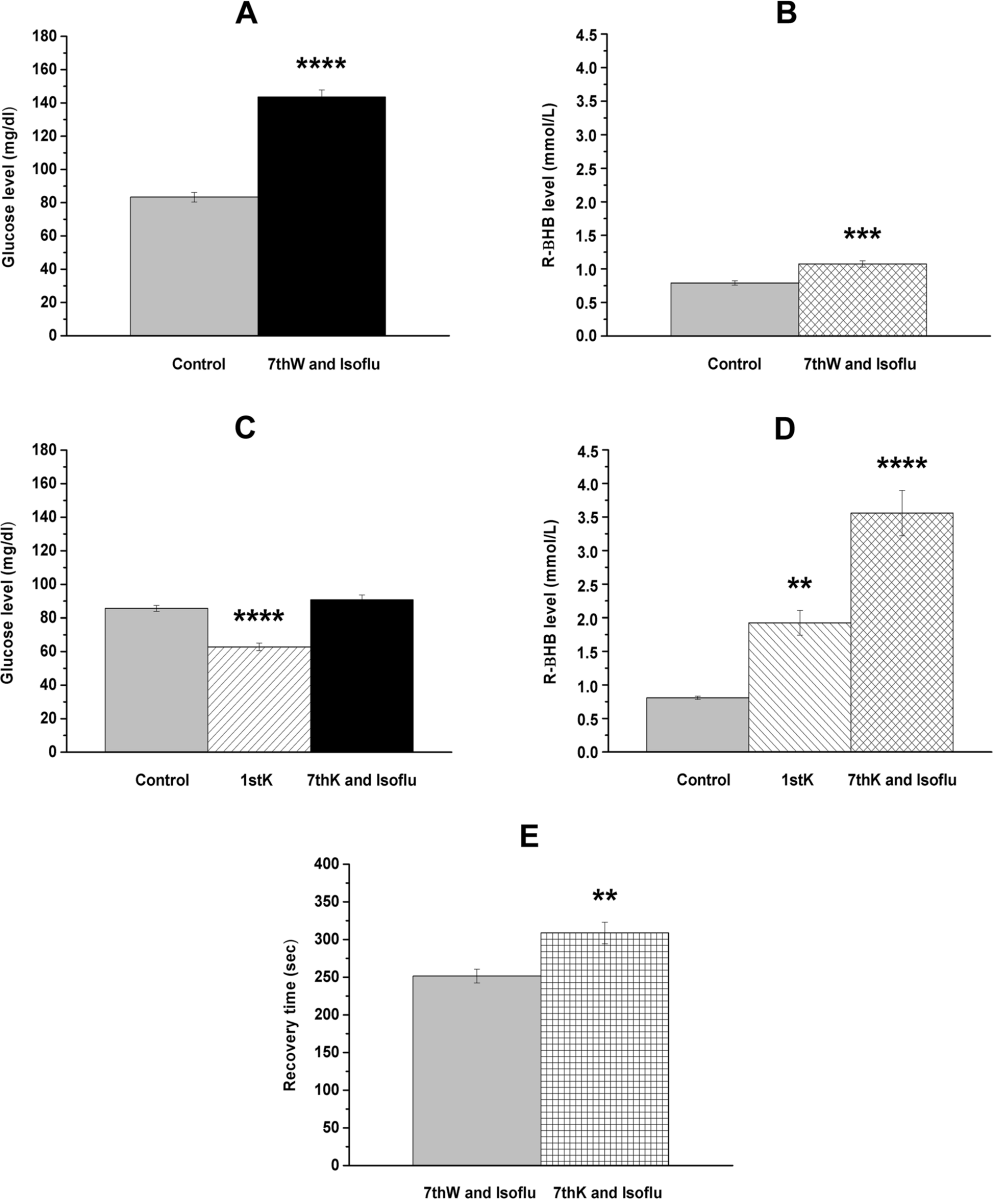
KEMCT-evoked influences on isoflurane anesthesia-generated changes in blood glucose and R-βHB levels and recovery time. Blood glucose and R-βHB levels significantly increased after isoflurane-evoked anesthesia (7^th^ water gavage + isoflurane anesthesia: 7thW and Isoflu; group 1) (**A** and **B**). KEMCT (mix of ketone ester/KE and medium chain triglyceride/MCT oil in a 1:1 ratio) treatment for 7 days abolished the isoflurane-generated increase in blood glucose level (7^th^ KEMCT gavage + isoflurane anesthesia: 7thK and Isoflu; group 2) (**C**). After both 1^st^ KEMCT treatment (1stK) alone (without anesthesia) and 7^th^ KEMCT treatment combined with isoflurane anesthesia (7thK and Isoflu; group 2), blood R-βHB levels were increased (**D**). In KEMCT treated animals (7thK and Isoflu; group 2) the time required for recovery from isoflurane-evoked anesthesia significantly increased, compared to water gavaged and isoflurane-anesthetized animals (7thW and Isoflu; group 1) (**E**). Abbreviations: 7thK and Isoflu, 7^th^ KEMCT gavage and isoflurane anesthesia; 7thW and Isoflu, 7.^th^ water gavage and isoflurane anesthesia; **p < 0.01; ***p < 0.001; ****p < 0.0001

Significant increase in both blood glucose (*p* < 0.0001) and blood R-βHB level (*p* = 0.0005) were generated by isoflurane-evoked anesthesia, compared to control (group 1) (Fig. 1A and 1B, respectively; Table 1). After first KEMCT treatment (group 2), blood level of glucose and R-βHB decreased *(p* < 0.0001) and increased (*p* < 0.0033), respectively, compared to control (Fig. 1C and 1D; Table 1). We demonstrated that 7^th^ KEMCT gavage abolished the isoflurane anesthesia-evoked increase in blood glucose level (compared to control, *p* = 0.2805) (Fig. 1A and 1C; Table 1). In other words, the KEMCT was able to maintain the normal (control) glucose level under isoflurane anesthesia-generated circumstances preventing the glucose level elevating effect of isoflurane. Moreover, the last (7^th^) KEMCT gavage significantly increased the level of blood R-βHB after isoflurane anesthesia (*p* < 0.0001) (Fig. 1D; Table 1). KEMCT-generated effects on blood glucose (after 1^st^ gavage) and R-βHB (after 1^st^ gavage and 7^th^ gavage combined with isoflurane anesthesia) levels were similar to our previous results (in which experiments, isoflurane anesthesia was not used in combination with EKSs) [27].

**Table 1:** Effect of KEMCT treatment on isoflurane anesthesia-evoked changes in blood R-βHB and glucose levels

**Treatments**	**Glucose** (mg/dl)	**R-βHB** (mmol/l)
**Water gavage (3 g/kg)** (Group 1; Fig. 1A and B)		
** Control** (measured on the last day of the adaptation period)	83.33 ± 2.867	0.79 ± 0.034
** After 7**^**th**^** gavage and isoflurane anesthesia** (7thW and Isoflu)	143.58 ± 4.177	1.08 ± 0.048
Compared to Control (significance level/*p* value)	****/ < 0.0001	***/0.0005
**KEMCT gavage (3 g/kg)** (Group 2; Fig. 1C and D)		
** Control** (measured on the last day of the adaptation period)	85.58 ± 1.786	0.81 ± 0.023
** After 1**^**st**^** gavage**	62.83 ± 2.306	1.93 ± 0.182
Compared to Control (significance level/*p* value)	****/ < 0.0001	**/ < 0.0033
** After 7**^**th**^** gavage and isoflurane anesthesia** (7thK and Isolfu)	90.75 ± 2.850	3.56 ± 0.339
Compared to Control (significance level/*p* value)	ns/0.2805	****/ < 0.0001

Abbreviations: 7thK and Isolfu, 7^th^ KEMCT gavage and isoflurane anesthesia; 7thW and Isoflu, 7^th^ water gavage and isoflurane anesthesia; KEMCT (mix of ketone ester/KE and medium chain triglyceride/MCT oil in a 1:1 ratio); ns, non-significant

^**^p < 0.01

^***^p < 0.001

^****^p < 0.0001

Similar to our previous studies [18, 27, 28] body weight of animals did not change significantly after water (group 1) and KEMCT (group 2) treatment, compared to control (control/water treated, group 1: 318.6 ± 2.69 g/319.7 ± 1.66 g, *p* = 0.9263; control/KEMCT treated, group 2: 319.8 ± 2.45 g/312.9 ± 2.52 g, *p* = 0.0963). 

### Effect of KEMCT gavage on recovery time 

Time required for recovery (recovery time) from isoflurane-evoked anesthesia significantly increased in KEMCT treated animals (group 2), compared to water gavaged animals (group 1) (water gavaged/KEMCT gavaged: 251.6 ± 9.14 s/308.8 ± 14.32 s, *p* = 0.0028) (Fig. 1E).

## Discussion

In this study, we first demonstrated that isoflurane anesthesia-evoked increase in blood glucose level was abolished by administration of KEMCT. Moreover, we extended our previous results on EKSs-generated effects in WAG/Rij rats showing that KEMCT treatment significantly increased the time required for recovery from isoflurane-induced anesthesia.

It has been suggested that ketosis may generate an increase in adenosine level [31] and modulation of sleep and sleep-like processes [16, 32]. Moreover, it was demonstrated that adenosine has a role in the isoflurane-generated anesthetic influence [32, 33]. For example, isoflurane is able to activate A1Rs [34] and infusion of adenosine decreased the requirement for isoflurane during surgery [35]. A non-selective antagonist of adenosine receptors theophylline reversed the cerebral effects of isoflurane [36] and caffeine accelerated the recovery from isoflurane anesthesia in humans and mouse [20, 37, 38]. It was also demonstrated that a selective A1R antagonist DPCPX (8-cyclopentyl-1,3-dipropylxanthine) decreased, whereas a selective adenosine A2A receptor (A2AR) antagonist ZM 241,385 (4-(2-[7-amino-2-(2-furyl)[1,2,4]triazolo[2,3-a][1,3,5]triazin-5-ylamino]ethyl)phenol) did not shorten the time required to recovery from isoflurane anesthesia (resumption of righting) [20]. Moreover, it was also demonstrated that administration of an A1R agonist N-p-sulfophenyl adenosine increased the time required for recovery from isoflurane-induced anesthesia in mice may be via A1Rs [19]. Thus, based on results above, KEMCT-evoked ketosis may be able to increase the time required to recover from isoflurane-induced anesthesia (Fig. 1E) through A1Rs. It was also demonstrated that a selective A2AR antagonist preladenant accelerated the recovery from isoflurane anesthesia in rats [39], suggesting that the role of A2ARs in KEMCT-generated effects on recovery time is also possible. However, our results strengthen the previous suggestion that inhibition of both A1Rs and A2ARs may be an efficient therapeutic tool for promoting emergence from inhalational anesthesia [40].

It has been demonstrated previously that administration of EKSs by intragastric gavage can increase blood ketone body (e.g. R-βHB) levels [10, 11, 29] (Fig. 1D). Increased blood level of ketone bodies under general (e.g. isoflurane) anesthesia was demonstrated in patients due to surgical stress and/or preoperative fasting [41, 42], and it was suggested that general anesthesia was likely safe for patients in ketosis [42, 43]. In this study, moderate, but significant increase in R-βHB level was measured several min after recovery from isoflurane-induced anesthesia (Fig. 1B) without surgical stress, fasting or administration of ketogenic compounds. These results suggest that KEMCT treatment could generate increase in blood R-βHB level on the 7^th^ day of KEMCT administration combined with isoflurane anesthesia (Fig. 1D), which KEMCT-induced effect may be slightly enhanced by isoflurane anesthesia-generated influence on blood βHB level. 

Hyperglycemia and insulin resistance (impaired insulin sensitivity) may be in association with immunosuppression, infectious complications, cardiovascular problems, increased risk in neurocognitive dysfunction and ischemic brain damage, and could worsen prognosis and mortality in surgical patients [6–9]. Insulin resistance and hyperglycemia may result from surgery-evoked stress [6]. However, isoflurane anesthesia further exacerbates the surgical stress-provoked hyperglycemic reaction and impairs glucose tolerance and hyperglycemia without surgical stress [5, 44–46]. This is likely mediated through isoflurane-evoked facilitation of opening of ATP-sensitive potassium channels in pancreatic β-cells (resulting decrease in release of insulin and glucose utilization) [46, 47]. It has also been concluded that activation of ATP-sensitive potassium channels by isoflurane in pancreatic β-cells could impair both insulin secretion and glucose tolerance resulting hyperglycemia-generated decrease in alleviating influences of isoflurane [47, 48]. Thus, under isoflurane-evoked anesthesia, continuous monitoring and tight control of blood glucose level and perioperative maintenance of normoglycemia may be necessary to prevent the hyperglycemia-generated neurological damage [49, 50]. Strategies to mitigate isoflurane-induced metabolic derangement may involve IV fluid and insulin administration [6, 51], but a rationale may also exist for EKSs (as an adjuvant therapy). This treatment may be helpful in stabilizing glycemia (Fig. 1C) for the anesthesiologists, and perhaps for the neuroprotective effect of ketosis [52]. In relation to putative mechanism of action, EKSs, such as KE not only increase blood ketone body level, but also decrease both blood glucose and insulin level [53], suggesting that EKS-induced ketosis can increase insulin sensitivity [54]. Moreover, it has also been demonstrated that activation of A1Rs may increase insulin sensitivity [21, 22], overexpression of A1Rs could protect from insulin resistance in mice [55], whereas impaired glucose tolerance and insulin sensitivity was detected in A1R KO mice [56]. Moreover, it was also demonstrated that not only direct facilitation (opening) of ATP sensitive potassium channels, but also A1R-evoked activation of ATP sensitive potassium channels may contribute to the isoflurane anesthesia-generated cardioprotection [48, 57, 58]. Consequently, as ketosis (βHB) may increase adenosine level [31], KEMCT-generated ketosis could modulate not only the time required for recovery from isoflurane-induced anesthesia and effect of isoflurane on blood glucose level, but also alleviating influences (e.g. cardioprotective effect) of isoflurane through A1Rs. 

One limitation of our study is that we narrowed our focus on the putative adenosinergic mechanism of KEMCT-generated effects on isoflurane anesthesia-evoked influences. However, it has been demonstrated that administration of the KEMCT can generate ketosis [18, 27, 28] (Fig. 1D), which may increase adenosine levels [31]. Enhanced adenosine levels can modulate the ketone supplements-evoked influences on (i) sleep (sleep-like) effects [16, 32], (ii) isoflurane-generated anesthesia [32, 33, 40] and (iii) insulin sensitivity [21, 22] likely through A1Rs. Thus, we could propose that the adenosinergic system may be one of the main factors by which KEMCT can exert its effects on isoflurane anesthesia. Moreover, it is likely that there are multiple factors, which can contribute to KEMCT-evoked effects such as the delay in emergence from anesthesia. For example, another potential factor may be the respiratory changes induced by ketosis or the mild acidosis caused by an increase in βHB levels after administration of EKSs [13]. Changes in respiration play a key role in elimination of inhalational anesthetics [59]. Thus, if the animal respiration was changed during KEMCT-induced ketosis, the elimination and the time required for recovery from isoflurane anesthesia will be altered. However, new studies are needed to explore different factors (e.g. changes in both activity of A1Rs and respiratory physiology), by which EKSs may be able to alter isoflurane anesthesia-generated effects.

## Conclusion

EKSs-evoked ketosis can modulate anesthesia-generated effects, and these modulatory influences of ketosis may be clinically and surgically relevant. Indeed, the EKS formulation given as KEMCT abolished the isoflurane-induced increase in blood glucose level whereas increased the time required for recovery from isoflurane-induced anesthesia. The last result suggests that administration of EKSs may modulate the requirement for isoflurane during surgery. Moreover, isoflurane anesthesia-induced increase in blood glucose level may decrease neuroprotective effects of isoflurane, thus, administration of EKSs (as an adjuvant therapy) can preserve and prevent some isoflurane-evoked alleviating influences and side effects, respectively. However, the effect of ketosis on alteration of blood glucose level under isoflurane- and, theoretically, other anesthetics-evoked anesthesia should be taken into account by anesthesiologists. For example, considerations should be taken when patients may be in self-induced ketosis due to its use as a medical therapy (epilepsy) or as a required fasting prior to anesthesia administration associated with medical procedures. Monitoring of not only blood ketone body level, but also glucose level pre-, intra-, and postoperatively in humans undergoing isoflurane anesthesia and maintenance of proper blood glucose level may be crucial to avoid potential harmful metabolic changes and their pathological consequences generated by interactions between EKSs-, isoflurane-, and other drugs-evoked effects. These results support the need for further male and female animal studies to demonstrate the exact mechanism of action of EKSs on isoflurane-generated increase in blood glucose level and recovery time from anesthesia. Furthermore, considering the expanding use of nutritional and supplemental ketosis additional studies in human subjects of different genders under normal and pathological conditions are needed to understand the implications of these findings.

## Data Availability

The data used and/or analyzed during the current study available from the corresponding author on reasonable request.

## Abbreviations

A1R: Adenosine A1 receptor
A2AR: Adenosine A2A receptor
EKS: Exogenous ketone supplement
i.p.: Intraperitoneal
KE: (ketone ester) 1,3 butanediol-acetoacetate diester
KEMCT: (mix of KE and MCT oil)
KS: Ketone salt
MCT: Medium chain triglyceride
R-βHB: R-beta-hydroxybutyrate
WAG/Rij: Wistar Albino Glaxo/Rijswijk

## References

[1] Franks NP (2008). General anaesthesia: from molecular targets to neuronal pathways of sleep and arousal. Nat Rev Neurosci.

[2] Hao X, Ou M, Zhang D, Zhao W, Yang Y, Liu J, Yang H, Zhu T, Li Y, Zhou C (2020). The effects of general anesthetics on synaptic transmission. Curr Neuropharmacol.

[3] Deng J, Lei C, Chen Y, Fang Z, Yang Q, Zhang H, Cai M, Shi L, Dong H, Xiong L (2014). Neuroprotective gases - fantasy or reality for clinical use?. Prog Neurobiol.

[4] Lattermann R, Schricker T, Wachter U, Georgieff M, Goertz A (2001). Understanding the mechanisms by which isoflurane modifies the hyperglycemic response to surgery. Anesth Analg.

[5] Saha JK, Xia J, Grondin JM, Engle SK, Jakubowski JA (2005). Acute hyperglycemia induced by ketamine/xylazine anesthesia in rats: mechanisms and implications for preclinical models. Exp Biol Med (Maywood).

[6] Ljungqvist O, Jonathan E (2012). Rhoads lecture 2011: Insulin resistance and enhanced recovery after surgery. J Parenter Enteral Nutr.

[7] Puskas F, Grocott HP, White WD, Mathew JP, Newman MF, Bar-Yosef S (2007). Intraoperative hyperglycemia and cognitive decline after CABG. Ann Thorac Surg.

[8] Turina M, Miller FN, Tucker CF, Polk HC (2006). Short-term hyperglycemia in surgical patients and a study of related cellular mechanisms. Ann Surg.

[9] Warner DS, Gionet TX, Todd MM, McAllister AM (1992). Insulin-induced normoglycemia improves ischemic outcome in hyperglycemic rats. Stroke.

[10] Ari C, Kovács Z, Juhasz G, Murdun C, Goldhagen CR, Koutnik AM, Poff AM, Kesl SL, D'Agostino D (2016). Exogenous ketone supplements reduce anxiety-related behavior in Sprague-Dawley and Wistar Albino Glaxo/Rijswijk rats. Front Mol Neurosci.

[11] D'Agostino DP, Pilla R, Held HE, Landon CS, Puchowicz M, Brunengraber H, Ari C, Arnold P, Dean JB (2013). Therapeutic ketosis with ketone ester delays central nervous system oxygen toxicity seizures in rats. Am J Physiol Regul Integr Comp Physiol.

[12] Hashim SA, VanItallie TB (2014). Ketone body therapy: from the ketogenic diet to the oral administration of ketone ester. J Lipid Res.

[13] Stubbs BJ, Cox PJ, Evans RD, Santer P, Miller JJ, Faull OK, Magor-Elliott S, Hiyama S, Stirling M, Clarke K (2017). On the metabolism of exogenous ketones in humans. Front Physiol.

[14] Allen CN (2008). Circadian rhythms, diet, and neuronal excitability. Epilepsia.

[15] Constantinides C, Murphy K (2016). Molecular and integrative physiological effects of isoflurane anesthesia: The paradigm of cardiovascular studies in rodents using magnetic resonance imaging. Front Cardiovasc Med.

[16] Hallböök T, Lundgren J, Rosén I (2007). Ketogenic diet improves sleep quality in children with therapy-resistant epilepsy. Epilepsia.

[17] Ari C, Kovács Z, Murdun C, Koutnik AP, Goldhagen CR, Rogers C, Diamond D, D'Agostino DP (2018). Nutritional ketosis delays the onset of isoflurane induced anesthesia. BMC Anesthesiol.

[18] Kovács Z, Brunner B, D'Agostino DP, Ari C (2020). Inhibition of adenosine A1 receptors abolished the nutritional ketosis-evoked delay in the onset of isoflurane-induced anesthesia in Wistar Albino Glaxo Rijswijk rats. BMC Anesthesiol.

[19] Gettys GC, Liu F, Kimlin E, Baghdoyan HA, Lydic R (2013). Adenosine A(1) receptors in mouse pontine reticular formation depress breathing, increase anesthesia recovery time, and decrease acetylcholine release. Anesthesiology.

[20] Van Dort CJ, Baghdoyan HA, Lydic R (2009). Adenosine A(1) and A(2A) receptors in mouse prefrontal cortex modulate acetylcholine release and behavioral arousal. J Neurosci.

[21] Dhalla AK, Wong MY, Voshol PJ, Belardinelli L, Reaven GM (2007). A1 adenosine receptor partial agonist lowers plasma FFA and improves insulin resistance induced by high-fat diet in rodents. Am J Physiol Endocrinol Metab.

[22] Töpfer M, Burbiel CE, Müller CE, Knittel J, Verspohl EJ (2008). Modulation of insulin release by adenosine A1 receptor agonists and antagonists in INS-1 cells: the possible contribution of 86Rb+ efflux and 45Ca2+ uptake. Cell Biochem Funct.

[23] Leung LS, Luo T, Ma J, Herrick I (2014). Brain areas that influence general anesthesia. Prog Neurobiol.

[24] Wang L, Holland L, Fong R, Khokhar S, Fox AP, Xie Z (2019). A pilot study showing that repeated exposure to stress produces alterations in subsequent responses to anesthetics in rats. PLoS ONE.

[25] Coenen AM, Van Luijtelaar EL (2003). Genetic animal models for absence epilepsy: a review of the WAG/Rij strain of rats. Behav Genet.

[26] Ari C, Murdun C, Koutnik AP, Goldhagen CR, Rogers C, Park C, Bharwani S, Diamond DM, Kindy MS, D'Agostino DP, Kovács Z (2019). Exogenous ketones lower blood glucose level in rested and exercised rodent models. Nutrients.

[27] Ari C, Murdun C, Goldhagen C, Koutnik AP, Bharwani SR, Diamond DM, Kindy M, D'Agostino DP, Kovacs Z (2020). Exogenous ketone supplements improved motor performance in preclinical rodent models. Nutrients.

[28] Kovács Z, Brunner B, D'Agostino DP, Ari C (2021). Age- and sex-dependent modulation of exogenous ketone supplement-evoked effects on blood glucose and ketone body levels in Wistar Albino Glaxo Rijswijk Rats. Front Neurosci.

[29] Kovács Z, D'Agostino DP, Dobolyi A, Ari C (2017). Adenosine A1 receptor antagonism abolished the anti-seizure effects of exogenous ketone supplementation in Wistar Albino Glaxo Rijswijk rats. Front Mol Neurosci.

[30] Kovács Z, D'Agostino DP, Ari C (2018). Anxiolytic effect of exogenous ketone supplementation is abolished by adenosine A1 receptor inhibition in Wistar Albino Glaxo/Rijswijk rats. Front Behav Neurosci.

[31] Sharma AK, Rani E, Waheed A, Rajput SK (2015). Pharmacoresistant epilepsy: A current update on non-conventional pharmacological and non-pharmacological interventions. J Epilepsy Res.

[32] Porkka-Heiskanen T, Strecker RE, Thakkar M, Bjorkum AA, Greene RW, McCarley RW (1997). Adenosine: a mediator of the sleep-inducing effects of prolonged wakefulness. Science.

[33] Tung A, Mendelson WB (2004). Anesthesia and sleep. Sleep Med Rev.

[34] Tas PW, Eisemann C, Roewer N (2003). The volatile anesthetic isoflurane suppresses spontaneous calcium oscillations in vitro in rat hippocampal neurons by activation of adenosine A1 receptors. Neurosci Lett.

[35] Segerdahl M, Ekblom A, Sandelin K, Wickman M, Sollevi A (1995). Peroperative adenosine infusion reduces the requirements for isoflurane and postoperative analgesics. Anesth Analg.

[36] Roald OK, Forsman M, Steen PA (1990). Partial reversal of the cerebral effects of isoflurane in the dog by theophylline. Acta Anaesthesiol Scand.

[37] Fong R, Wang L, Zacny JP, Khokhar S, Apfelbaum JL, Fox AP, Xie Z (2018). Caffeine accelerates emergence from isoflurane anesthesia in humans: A randomized, double-blind, crossover study. Anesthesiology.

[38] Wang Q, Fong R, Mason P, Fox AP, Xie Z (2014). Caffeine accelerates recovery from general anesthesia. J Neurophysiol.

[39] Fong R, Khokhar S, Chowdhury AN, Xie KG, Wong JH, Fox AP, Xie Z (2017). Caffeine accelerates recovery from general anesthesia via multiple pathways. J Neurophysiol.

[40] Kelz MB, García PS, Mashour GA, Solt K (2019). Escape from oblivion: neural mechanisms of emergence from general anesthesia. Anesth Analg.

[41] Murakawa T, Satoh Y, Kudo M, Kudo T, Matsuki A (1995). Arterial plasma keton body levels during isoflurane anesthesia and surgery. Masui.

[42] Ohkawa H, Iwakawa T, Ohtomo N, Kitayama M, Miyahara A, Ishihara H, Matsuki A (1993). Clinical study on intraoperative hyperketonemia in non-diabetic surgical patients under general anesthesia. Masui.

[43] Soysal E, Gries H, Wray C (2016). Pediatric patients on ketogenic diet undergoing general anesthesia-a medical record review. J Clin Anesth.

[44] Behdad S, Mortazavizadeh A, Ayatollahi V, Khadiv Z, Khalilzadeh S (2014). The effects of propofol and isoflurane on blood glucose during abdominal hysterectomy in diabetic patients. Diabetes Metab J.

[45] Horber FF, Krayer S, Miles J, Cryer P, Rehder K, Haymond MW (1990). Isoflurane and whole body leucine, glucose, and fatty acid metabolism in dogs. Anesthesiology.

[46] Tanaka T, Nabatame H, Tanifuji Y (2005). Insulin secretion and glucose utilization are impaired under general anesthesia with sevoflurane as well as isoflurane in a concentration-independent manner. J Anesth.

[47] Tanaka K, Kawano T, Tomino T, Kawano H, Okada T, Oshita S, Takahashi A, Nakaya Y (2009). Mechanisms of impaired glucose tolerance and insulin secretion during isoflurane anesthesia. Anesthesiology.

[48] Tanaka K, Kehl F, Gu W, Krolikowski JG, Pagel PS, Warltier DC, Kersten JR (2002). Isoflurane-induced preconditioning is attenuated by diabetes. Am J Physiol Heart Circ Physiol.

[49] Furnary AP, Zerr KJ, Grunkemeier GL, Starr A (1999). Continuous intravenous insulin infusion reduces the incidence of deep sternal wound infection in diabetic patients after cardiac surgical procedures. Ann Thorac Surg.

[50] Muoio DM, Newgard CB (2008). Mechanisms of disease: Molecular and metabolic mechanisms of insulin resistance and beta-cell failure in type 2 diabetes. Nat Rev Mol Cell Biol.

[51] Ittichaikulthol W, Lekprasert V, Pausawasdi S, Suchartwatnachai P (1997). Effect of intraoperative fluid on blood glucose level in neurosurgery. J Med Assoc Thai.

[52] Gambardella I, Ascione R, D'Agostino DP, Ari C, Worku B, Tranbaugh RF, Ivascu N, Villena-Vargas J, Girardi LN (2021). Systematic Review - Neuroprotection of ketosis in acute injury of the mammalian central nervous system: A meta-analysis. J Neurochem.

[53] Kashiwaya Y, Pawlosky R, Markis W, King MT, Bergman C, Srivastava S, Murray A, Clarke K, Veech RL (2010). A ketone ester diet increases brain malonyl-CoA and Uncoupling proteins 4 and 5 while decreasing food intake in the normal Wistar Rat. J Biol Chem.

[54] Sato K, Kashiwaya Y, Keon C, Tsuchiya N, King MT, Radda GK, Chance B, Clarke K, Veech RL (1995). Insulin, ketone bodies, and mitochondrial energy transduction. FASEB J.

[55] Dong Q, Ginsberg HN, Erlanger BF (2001). Overexpression of the A1 adenosine receptor in adipose tissue protects mice from obesity-related insulin resistance. Diabetes Obes Metab.

[56] Faulhaber-Walter R, Jou W, Mizel D, Li L, Zhang J, Kim SM, Huang Y, Chen M, Briggs JP, Gavrilova O, Schnermann JB (2011). Impaired glucose tolerance in the absence of adenosine A1 receptor signaling. Diabetes.

[57] Roscoe AK, Christensen JD, Lynch C (2000). Isoflurane, but not halothane, induces protection of human myocardium via adenosine A1 receptors and adenosine triphosphate-sensitive potassium channels. Anesthesiology.

[58] Tanaka K, Weihrauch D, Ludwig LM, Kersten JR, Pagel PS, Warltier DC (2003). Mitochondrial adenosine triphosphate-regulated potassium channel opening acts as a trigger for isoflurane-induced preconditioning by generating reactive oxygen species. Anesthesiology.

[59] Sakai EM, Connolly LA, Klauck JA (2005). Inhalation anesthesiology and volatile liquid anesthetics: focus on isoflurane, desflurane, and sevoflurane. Pharmacotherapy.

